# Altered miRNAs Expression Correlates With Gastroenteropancreatic Neuroendocrine Tumors Grades

**DOI:** 10.3389/fonc.2020.01187

**Published:** 2020-07-17

**Authors:** Elisabetta Cavalcanti, Vanessa Galleggiante, Sergio Coletta, Elisa Stasi, Marcello Chieppa, Raffaele Armentano, Grazia Serino

**Affiliations:** National Institute of Gastroenterology “S. de Bellis”, Research Hospital, Castellana Grotte, Italy

**Keywords:** miRNAs, neuroendocrine tumors, gastrointestinal tract, tumor grading, molecular markers, miR-96-5p, FoXO1

## Abstract

Gastroenteropancreatic neuroendocrine tumors (GEP-NETs) are rare and heterogeneous tumors that present a wide spectrum of different clinical and biological characteristics. Currently, tumor grading, determined by Ki-67 staining and mitotic counts, represents the most reliable predictor of prognosis. This time-consuming approach fails to reach high reproducibility standards thus requiring novel approaches to support histological evaluation and prognosis. In this study, starting from a microarray analysis of paraffin-embedded tissue specimens, we defined the miRNAs signature for poorly differentiated NETs (G3) compared to well-differentiated NETs (G1 and G2) consisting of 56 deregulated miRNAs. We identified 8 miRNAs that were expressed in all GEP-NETs grades but at different level. Among these miRNAs, miR-96-5p expression level was progressively higher from grade 1 to grade 3; inversely, its target FoxO1 expression decreased from grade 1 to grade 3. Our results reveal that the miRNAs expression profile of GEP-NET is correlated with the tumor grade, showing a potential advantage of miRNA quantification that could aid clinicians in the classification of common GEP-NETs subtypes. These findings could reliably support the histological evaluation of GEP-NETs paving the way toward personalized treatment approaches.

## Introduction

Neuroendocrine Tumors (NETs) are rare and heterogeneous tumors that present with a wide spectrum of different clinical and biological characteristics (1). In the last years, several studies have been conducted to gain a better understanding of NETs pathogenesis, and refined grading systems and classification according to the site of origin, cell types, and pathological features.

Regardless of the new classification (2010/2017 WHO), the low incidence, tumor heterogeneity, non-specific symptoms at presentation, undefined nomenclatures and classifications, NETs remain an unpredictable disease often difficult to be diagnosed, particularly when the disease is still at an early stage. In fact, the absence of pathognomonic symptoms makes the diagnosis difficult; they remain asymptomatic for years and are discovered only when they are already metastatic (2). NETs are commonly observed in the gastrointestinal tract, in the pancreas and in the lung; incidence rates have risen from 1.52 to 7.41 cases per 100,000 (3, 4). According to the latest WHO classification, NETs are classified by morphological characteristics and the assessment of proliferation in two main categories: well-differentiated (WD) and poorly differentiated (PD), also called G1/G2 and G3, respectively. Specifically, Ki-67 index and mitotic count were used to assess the grading (G1-G2-G3) (5, 6). According to WHO 2010, grade G1 and G2 are defined as neuroendocrine tumors (NETs) and G3 as neuroendocrine carcinoma (NEC3); they feature different prognoses, outcomes, and treatment approaches (7). Since well-differentiated, high-grade NETs clearly exist (mostly in the pancreas) and it were not considered a homogeneous entity, the WHO described a new classification in 2017 that discriminates the well-differentiated (low-grade, intermediate-grade, or high-grade) pNETs and poorly differentiated (high-grade) pancreatic NECs (pNECs) (8); but this term is currently limited to patients with pancreatic NETs (pNETs). A more recent classification was made to the histological and cytology pattern, in particular NECs are no longer graded, as they are recognized to be uniformly high grade by definition, but continue to be separated into small-and large-cell types (9, 10).

Recent studies have demonstrated that molecular markers could be added to morphologic evaluation for more accurate classification, especially in small biopsies where diagnostic materials may be limited. In our previous studies, we highlighted the importance of a better characterization of NETs grading to gain more reliable prognostic and therapeutic indications. In particular, we analyzed the role of programmed cell death ligand 1 (PD-L1) in GEP-NET: PD-L1 expression was significantly associated with a high-grade WHO classification (G3), becoming a new gold standard for G3 NET discrimination (11). Furthermore, pharmacological approaches using anti-PD-1 antibodies may become the choice for the treatment of G3 cases with a poor prognosis, while for G1/G2 cases anti-angiogenic drugs could be an excellent therapeutic choice, as demonstrated in another recent study by our group (12).

In NETs, the traditional cytotoxic drugs have shown limited efficacy, although their biological features have been characterized and can be therapeutically exploited. Hence, further investigations into the molecular basis of neuroendocrine tumors are needed. Among these potential novel diagnostic and therapeutic targets, microRNAs (miRNAs) represent a class of small and endogenous non-coding RNAs that regulate gene expression at post-transcriptional level, inhibiting the translation of specific mRNAs (13). Specifically, miRNAs can regulate various cellular processes including proliferation, migration, apoptosis, and differentiation (14). The role of miRNAs in cancer is well-established, and many studies have demonstrated their function in cancerogenesis and tumor aggressiveness (15, 16). Several studies have already underlined specific miRNA biomarkers in different types of cancer such as lung cancer (17), pancreatic cancer (18), liver cancer (19), colorectal cancer (20), gastric cancer (21), and esophageal cancer (22). Moreover, miRNAs dysregulation was also associated with tumor prognosis and therapy response.

In Gastroenteropancreatic Neuroendocrine Neoplasms (GEP-NETs), data on miRNAs expression are limited (23–25) and the main studies have been conducted in pNETs (26). Specific miRNAs signatures were able to discriminate pNETs from pancreatic ductal adenocarcinoma (27) and acinar pancreatic tumors (28), cystic forms of pNETs from other pancreatic cystic lesions (29). In small bowel NETs, especially those of the ileum, 29 deregulated miRNAs were identified in primary tumors including the upregulated miR-204-5p, miR-7-5p, and miR-375 that inhibits cell proliferation/induces apoptosis. Comparison between primary tumors with liver and lymph node metastases highlighted numerous deregulated miRNAs (30). High levels of circulating miR-21-5p and miR-22-3p and low levels of miR-10-5p were found in patients with metastatic small intestine neuroendocrine tumors, and there was a direct correlation between the levels of these miRNAs and overall survival (31).

In this study, starting from microarray analysis, we examined the global miRNAs expression profile of GEP-NET in different anatomic sites, correlating their expression with grading. For the first time we provide the specific miRNA signature for each GEP-NET grade identifying miRNAs differently expressed in grade 1 to grade 3. In particular, we showed that miR-96-5p expression levels was increased from grade 1 to grade 3, whereas its target FoxO1 decreased from grade 1 to grade 3. Our results support an important and unreported link between miRNA expression and GEP-NET grades. Our data support future studies focused on personalized pharmacological approach and offering a point of reference for the use of miRNA expression as target for GEP-NET grading using liquid biopsy specimens.

## Materials and Methods

### Patients Characteristics and Pathological Assessment

Ninety formalin-fixed and paraffin-embedded tissue specimens of NETs collected from January 2006 to January 2019 at the IRCCS “Saverio De Bellis” of Castellana Grotte (BA, Italy) were enrolled in this study after approval of the Ethics Committee of the “Istituto Tumori Giovanni Paolo II” (BA, Italy). The study was carried out according to the principles of the Declaration of Helsinki. The patients provided written informed consent. For all patients, we collected the following clinicopathological features: age, gender, primary site, tumor grade, metastasis, angioinvasion and lymphocytic infiltration (Table 1).

**Table 1:** Clinicopathologic features of 90 GEP-NET patients.

	**Tot**	**%**
**Gender**		
Men	48	53
Women	42	47
**Age, years**		
Median, range	62.7, (20–93)	
**Tumor site**		
Stomach	25	27.78
Small intestine	24	26.67
Liver	13	14.44
Pancreas	11	12.22
Colon-rectum	7	7.78
Appendix vermiform	7	7.78
Gallbladder	3	3.33
**Grade WHO classification**		
G1	62	68.89
G2	12	13.33
G3	16	17.78
**Angioinvasion**		
Absent	73	81.11
Present	17	18.89
**Lymphocytic infiltration**		
Absent	71	78.89
Present	19	21.11
**Lymph nodes metastasis**	3	3.3
**Treatments**	81	72.90

Formalin fixed paraffin embedded (FFPE) tissue sections stained with hematoxylin and eosin (H&E) was reviewed by two pathologists and a representative paraffin block from each specimen was chosen for immunohistochemistry (IHC) analysis. On H&E and PAS mucin-stained sections, the cytological characteristics of cells and the presence of ulcerations, perineural infiltration, vascular permeation necrosis, and lymph node metastasis were evaluated. All the cases were reviewed to confirm the diagnoses according to the WHO 2010 and to the last WHO 2017 only for p-NETs (8).

### miRNA Isolation

Total RNA, including small RNA fraction, was isolated from FFPE section of 5 μm-thickness using miRNeasy FFPE kit (Qiagen) according to the manufacturer's protocol including the treatment of sections with Deparaffinization Solution (Qiagen). Total RNA was then eluted in ribonuclease-free water. The RNA concentration was determined with the NanoDrop ND-2000 Spectrophotometer (Nanodrop Technologies).

### miRNA Microarray

The miRNA expression levels were assessed using SurePrint G3 Human Microarray Agilent Human 8x60K miRNA Microarray (Agilent Technologies) based on Sanger miRBase release 21, according to the manufacturer's instructions. Briefly, 100 ng of total RNA isolated from FFPE section of 18 NETs patients were firstly dephosphorylated with a calf intestine alkaline phosphatase treatment for 30 min at 37°C before labeling. Samples were diluted with Dimethyl sulfoxide (DMSO), denatured for 10 min at 100°C, and labeled using pCp-Cy3 in T4 RNA ligation buffer. The labeled RNA was hybridized, washed, stained, and scanned with an Agilent microarray scanner (G2565BA, Agilent). Microarray data analysis was performed using Agilent Feature Extraction Software 12.1 (Agilent) using default parameters.

Microarray data are available under accession number GSE135034 at the Gene Expression Omnibus (http://www.ncbi.nlm.nih.gov/geo/).

### Statistical Analyses and Bioinformatics

For microarray analysis, the raw expression signals were log-transformed, normalized, and filtered according to the median corrected signal of all the miRNAs with an intensity >100 (considered as expressed) and analyzed using Agilent Gene Spring GX 14.9 software. Probe sets were selected based on significant *P*-value and were adjusted to account for multiple testing using the Benjamini-Hochberg FDR method. To determine miRNAs that were differentially expressed between NETs grading, we applied a filter for FDR < 0.05 and a fold change of ± 2. Hierarchical clustering and principal component analyses (PCA) were created with Genesis software (http://www.genesis-softwareonline.com/) using average-linkage clustering method (32).

miRNA targets were predicted by means of miRBase 21.1 (33), TargetScan 7.1 (http://www.targetscan.org/vert_71/) (34), miRWalk 2.0 (http://zmf.umm.uni-heidelberg.de/apps/zmf/mirwalk2/) (35), miRDB (http://www.mirdb.org/) (36), and TarBase v.8 (http://carolina.imis.athena-innovation.gr/diana_tools/web/index.php?r=tarbasev8/index) (37) algorithms. Potential targets on the basis of overlapping results from the five algorithms, and selecting targets genes predicted by at least two of the algorithms. To assess biologic relationships among genes controlled by deregulated miRNAs, we used miRSystem ver. 20160513 (38) and miRPath v.3 (39) software. Statistical analysis was performed using GraphPad Prism statistical software release 5.0. Starting from the list of target genes for the eight deregulated miRNAs predicted by miRWalk, the gene ontology enrichment analysis was performed with Panther analysis tool (40).

Statistical differences between different conditions were assessed with two-tailed Student's *t*-test. All values are expressed as the mean ± SEM. Results were considered statistically significant at *p* < 0.05.

### Quantitative Real-Time PCR

Total RNA, including small RNA fractions, was reverse transcribed with the TaqMan Advanced miRNA cDNA Synthesis Kit (Thermo Fisher Scientific, MA, USA) following the manufacturer's protocol. Real-time RT-PCR for the quantification of a set of miRNAs (miR-96-5p, miR-7-5p, miR-130b-3p, miR-192-5p, and miR-194-5p, plus an endogenous control miR-26a-5p) was carried out with TaqMan Advanced miRNA assays and TaqMan Fast Advanced Master mix (Thermo Fisher Scientific, MA, USA). Real-time PCR amplification reactions were performed in 20 μl of final volume on a CFX96 System (Biorad Laboratories, CA, USA). Normalization was performed on the endogenous control miR-26a-5p, which has been found highly and equally expressed in microarray data.

### *In situ* Hybridization

*In situ* hybridization for miR-96-5p and *FoxO1* was performed on 6 μm paraffin sections with probes using double or single FAM-labeled locked nucleic acid (LNA) according to the manufacturer's instructions (Exiqon). Briefly, FFPE sections were deparaffinized in xylene and then rehydrated through an ethanol dilution series (from 100 to 70%). Then, sections were treated with Proteinase K at 37°C for 15 min and then washed with PBS. Labeled probes for miR-96-5p and *FoxO1* were denatured at 90°C for 4 min. Slides were incubated with the diluted probes in hybridization buffer at 57°C for 1 h. Stringent washes were performed with 5X saline sodium citrate (SSC), 1X SSC, and 0.2 SSC buffers at 57°C for 5 min. Slides were washed in PBS and mounted in medium containing DAPI (4′, 6-diamidino-2-phenylindole dihydrochloride) (Thermo Fisher Scientific). Fluorescent images of FAM and DAPI were taken at 488 and 358 nm, respectively, on a Nikon Eclipse Ti2 microscope (Nikon, Tokyo, Japan). Scramble probe was used as a negative control, and β-actin as positive control.

### IHC and IHC Evaluation

IHC analysis for FoxO1 protein was performed in the FFPE of 90 patients with NETs. Tumor sections of 4 μm were freshly cut and dried at 60°C for 30 min. IHC analysis was carried out in sections after deparaffinization for 30 min and then rehydration in grades of alcohol. Antigen retrieval was performed at 90°C for 20 min with Tris-borate-EDTA Buffer. To assess the FoxO1 staining employed for the present study, antibodies (clone EP927Y, Abcam, at 1:250 dilution) were evaluated on the NETs, using an automated autostainer (cat. K5007, Dako, Glostrup, Denmark). The Real Envision DAB Substrate Kit (DAKO) was used according to the manufacturer's instructions. FoxO1 expression was scored for all staining patterns, according to both the staining intensity and the percentage of positively stained cells, by two independent, blinded pathologists. The proportion of FoxO1-positive cells was estimated as the percentage of total tumor cells; tumor cells typically showed cytoplasmatic staining with a variable nuclear staining component. FoxO1 expression was scored as 0: (no staining) negative; 1: weak expression, but weaker than the positive control, staining in <5% of tumor cells; 2: moderate expression in > 5% of tumor cells; and 3: strong more than positive control staining in >5% of the tumor cells. For data assessment, our cases were considered positive for FoxO1 expression only if they had scores of 2+ or 3+.

Ki67 IHC was carried out again on all cases on paraffin-embedded sections using an Ab anti-Ki67 diluted 1:100 (Mib-1; DAKO) following the manufacturer's instructions. All cases were reviewed and re-evaluated and then assigned a precise proliferation index number that encompassed the amplitude limit of the 2010/2017 WHO range.

## Results

### Clinicopathologic Features

Table 1 summarizes the main clinicopathological characteristics of the 90 patients enrolled in the study. Median age of patients was 62.7 years (range: 20–93): 42 females (47%) and 48 males (53%). The most common primary site in our cohort of GEP-NET patients was the stomach (27.78%), followed by the small intestine (26.67%), liver (14.4%), pancreas (12.2%), colon-rectum (7.78%), appendix (7.78%), and gallbladder (3.33%). According to 2010/2017 WHO, the 90 cases analyzed were classified as follows: 62 grade 1 (68.89%), 12 grade 2 (13.33%), and 16 grade 3 (17.78%) Lymph node metastases and visceral peritoneum invasion were observed in 3 (2.7%) NET G1 (head of the pancreas and ileum). Curative treatments, such as surgery or endoscopic resection, were performed in 72.9% of patients (*n* = 81).

### miRNA Expression Profile in Patients With GEP-NETs

The role of miRNA expression in GEP-NETs tumor grades has not been well-explored. In order to determine whether the analysis of miRNA expression profiles may discriminate between the 3 GEP-NETs tumor grades, we performed miRNA global expression profile in 18 GEP-NETs patients (7 G1, 5 G2, and 6 G3).

**Figure 1:**
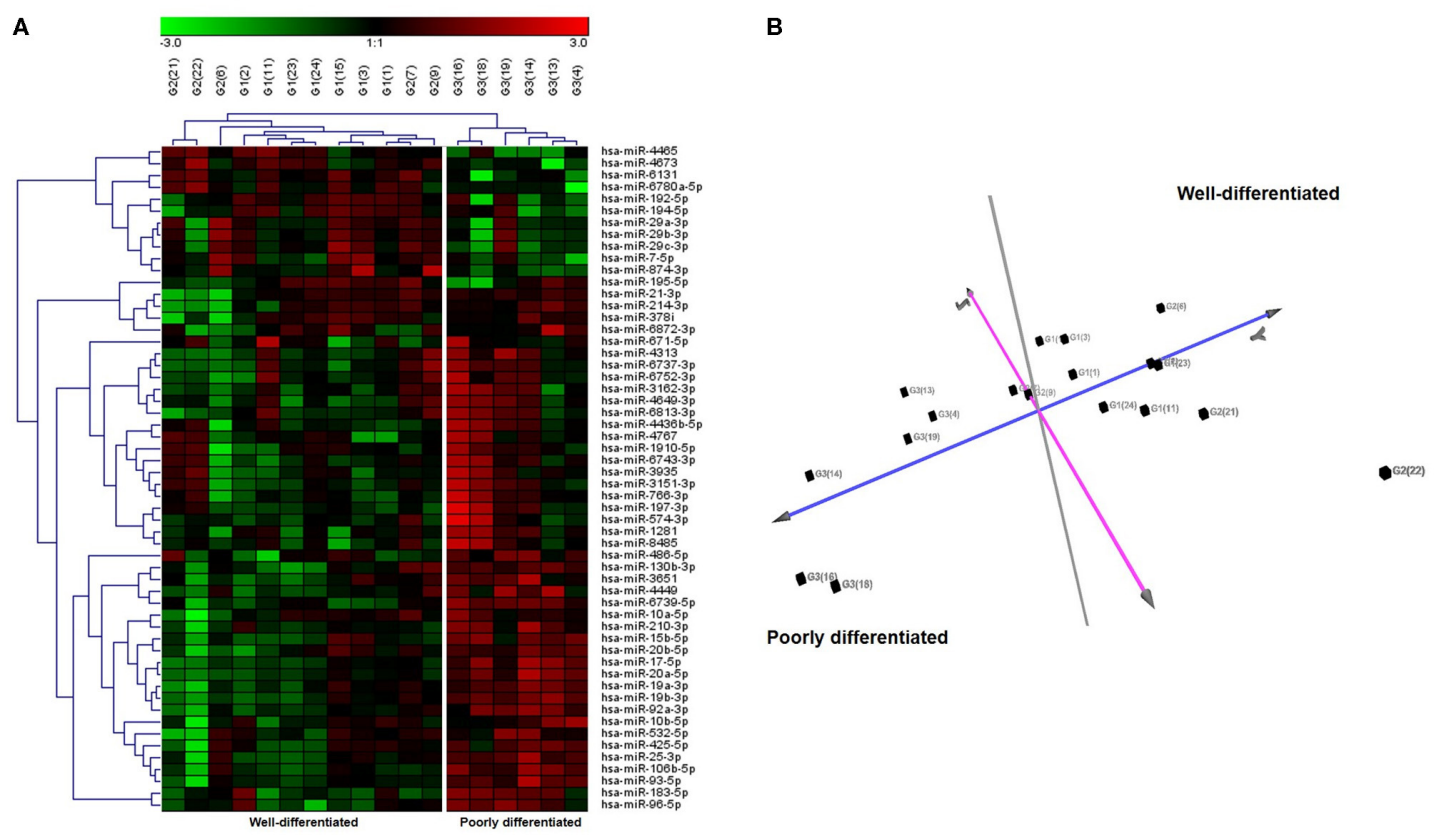
Unsupervised hierarchical clustering and principal component analysis (PCA) of 18 GEP-NETs patients by miRNAs expression profiles. **(A)** Hierarchical clustering using the 56 significantly deregulated miRNAs (FDR ≤ 0.05 and fold change threshold >2) discriminating patients with grading G3 (poorly differentiated NETs) vs. G1 and G2 (well-differentiated NETs) analyzed by microarray. Each column represents a sample and each row a miRNA. Red color indicates high expression and green low expression, according to the legend at the top. miRNA symbols are specified on the right side. Based on miRNA expression, we identified two principal clusters. **(B)** PCA based on the expression of differentially expressed miRNA in all samples. PCA displayed evident clustering and confirmed the separation between poorly differentiated NETs and well-differentiated NETs.

Differential expression analysis (FDR ≤ 0.05 and fold change threshold >2) between G3 vs. G1 patients identified 113 deregulated miRNAs (72 upregulated and 41 downregulated), instead the comparison between patients with grading G3 (poorly differentiated NETs) vs. G1 and G2 (well-differentiated NETs) showed 56 deregulated miRNAs (44 upregulated and 12 downregulated) (Supplementary Table 1). Surprisingly, there were significant differences in miRNAs expression between G1 and G2 NETs patients (50 deregulated miRNAs, 25 upregulated, and 25 downregulated). Unsupervised hierarchical clustering analysis generated a tree showing two clearly separated groups, one for G1 and G2 and the other one for G3 patients (Figure 1A). Principal component analysis (PCA) further confirmed this separation (Figure 1B).

### NETs Tumor Grades Are Associated With Selected miRNAs Expression Level

**Figure 2:**
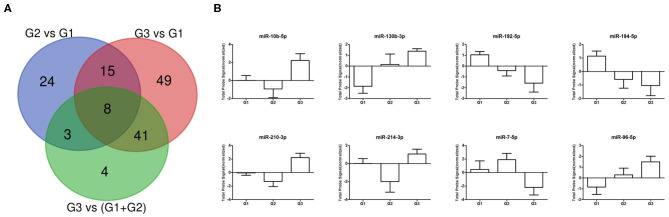
Differentially expressed miRNA across GEP-NETs grades. **(A)** Venn diagram showing the number of dysregulated miRNAs for each analyzed comparison. **(B)** Normalized probe signals are plotted for eight commonly expressed miRNAs that were differentially expressed in all 3 grades.

**Figure 3:**
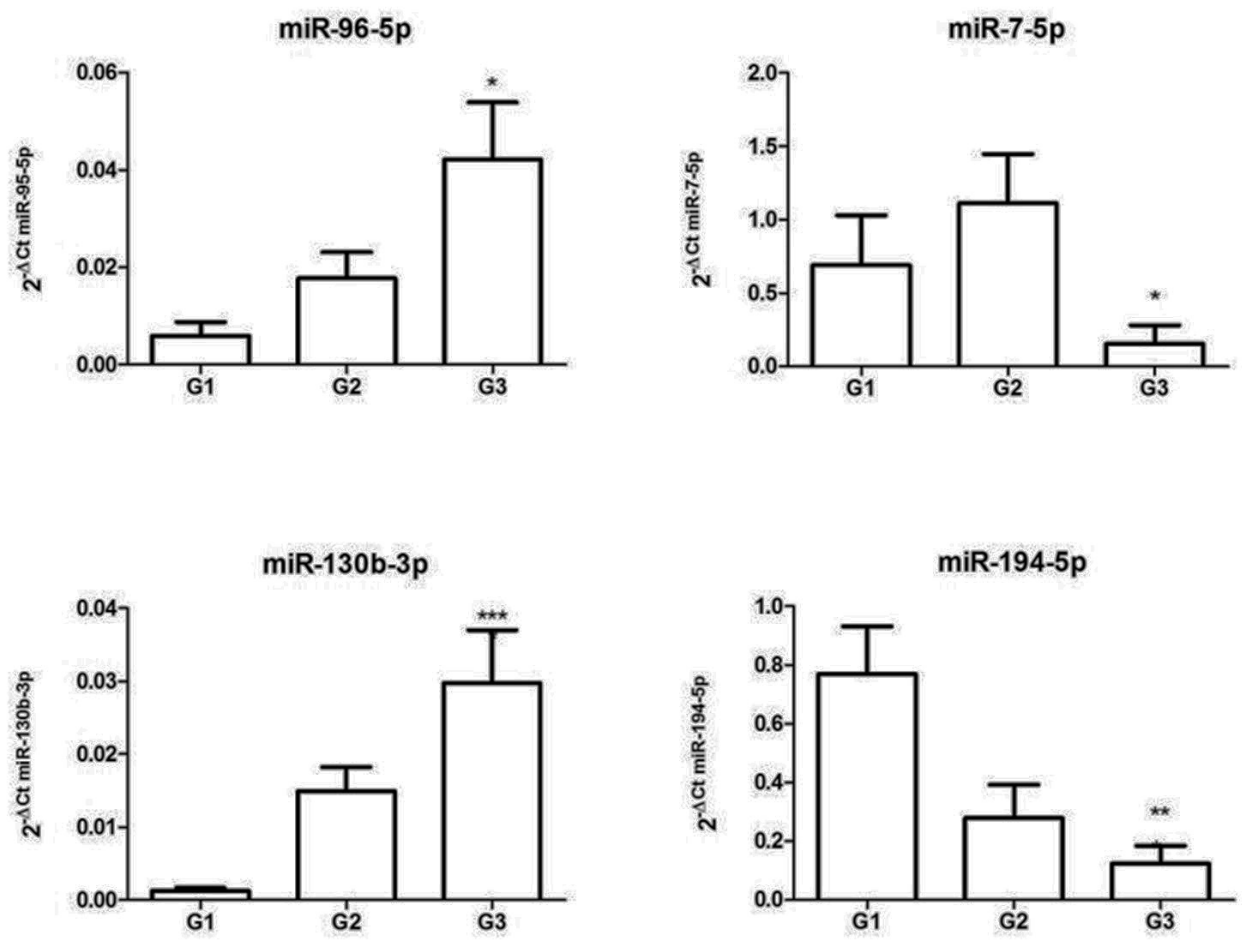
Validation of differentially expressed miRNAs. Expression levels of miR-96-5p, miR-7-5p, miR-130b-3p, and miR-194-5p from miRNAs isolated from a cohort of 24 GEP-NETs patients (8 G1, 8 G2, and 8 G3). Expression levels were quantified using qRT-PCR. The miRNA relative expressions were normalized to the expression of miR-26a-5p. The expression of all analyzed miRNAs was significantly modulated in all NETs tumor grades, confirming microarray data. The histograms represent the mean ± SEM. By ANOVA *p < 0.05, **p < 0.01, ***p < 0.001.

Starting from miRNAs expression data obtained in each comparison, we first focused our attention on miRNAs commonly expressed in all grades. The Venn diagram of grade-specific genes revealed that 8 miRNAs were expressed in all grades (Figure 2). Specifically, the miRNAs expressed in all grades were: hsa-miR-10b-5p, hsa-miR-130b-3p, hsa-miR-192-5p, hsa-miR-194-5p, hsa-miR-210-3p, hsa-miR-214-3p, hsa-miR-7-5p, and hsa-miR-96-5p. Moreover, the analysis of microarray data demonstrated that these 8 miRNAs had a different expression level in each grade (Figure 2). To validate microarray results, we performed quantitative real-time PCR (qRT-PCR) for miR-96-5p, miR-7-5p, miR-130b-3p, and miR-194-5p on miRNAs isolated from a cohort of 24 GEP-NETs patients (8 G1, 8 G2, and 8 G3). The expression of all analyzed miRNAs was significantly modulated in all NETs tumor grades confirming microarray data (Figure 3).

### *In silico* Analysis of miRNA Targets

To study the molecular mechanisms in which these miRNAs are involved, we performed a bioinformatic analyses to predict target genes of aforementioned eight deregulated miRNAs (hsa-miR-10b-5p, hsa-miR-130b-3p, hsa-miR-192-5p, hsa-miR-194-5p, hsa-miR-210-3p, hsa-miR-214-3p, hsa-miR-7-5p, and hsa-miR-96-5p). Based on bioinformatic analyses we found that deregulated miRNAs were mainly involved in several characteristic pathways of cancer. In particular, the identified miRNAs are involved in crucial pathways for cancer onset and progression, specifically Proteoglycans in cancer, Hippo signaling, Pathways in cancer, p53 signaling, FoxO signaling, HIF-1 signaling (Supplementary Table 2). Importantly, within these pathways, multiple miRNAs were predicted to regulate the same target genes, and one single miRNA could target several crucial genes. Moreover, Gene Ontology enrichment analysis demonstrated that the predicted gene targets of the eight deregulated miRNAs were significantly enriched in 313 biological processes (Supplementary Table 3). Using functional enrichment analysis, predicted target genes were categorized into several biological processes including regulation of cellular processes, regulation of cellular metabolic process, and regulation of cell communication.

### miR-96-5p Targets FoxO1 in GEP-NETs

**Figure 4:**
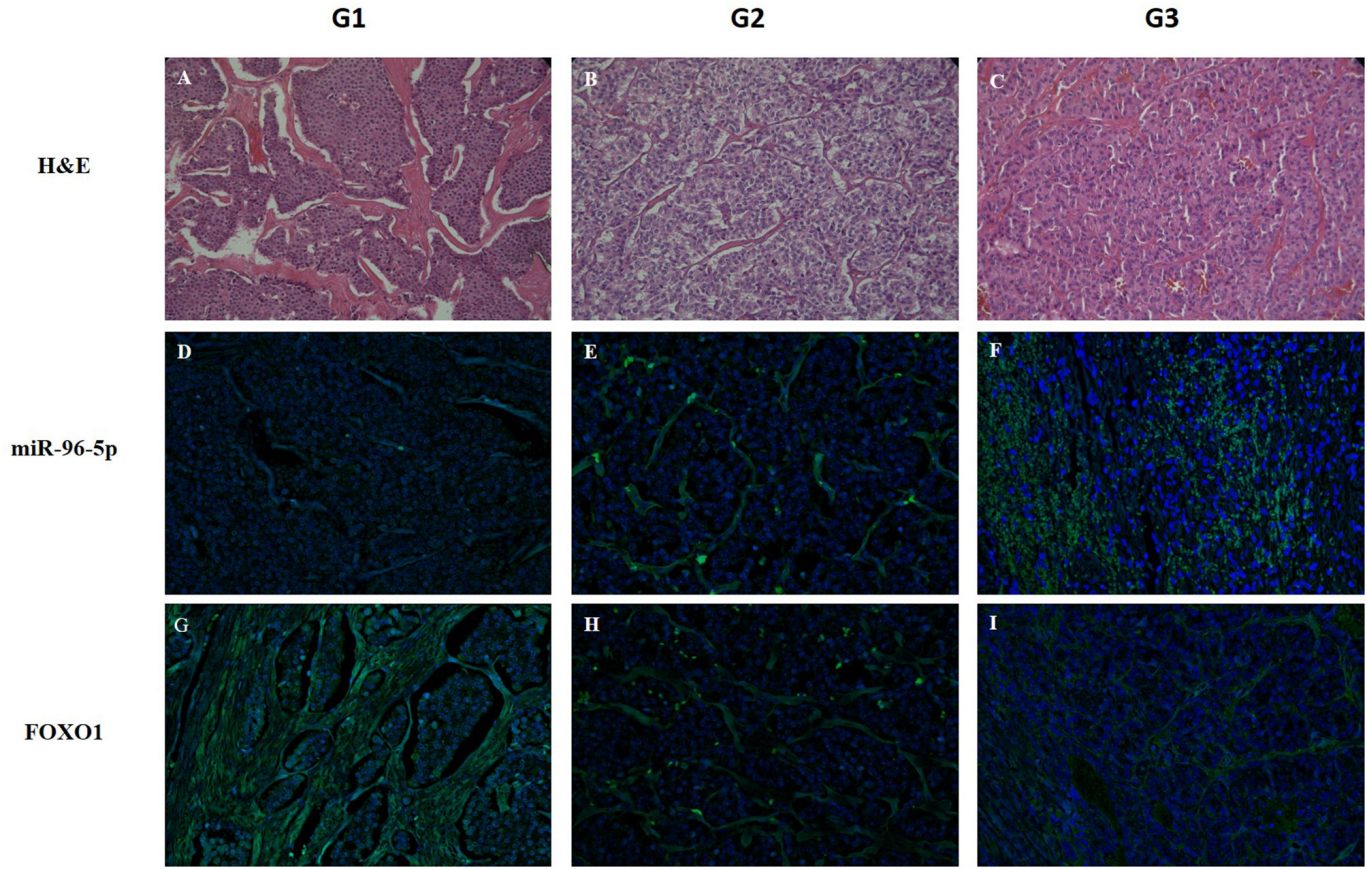
Detection of miR-96-5p and *FoxO1* mRNA by *in situ* hybridization. LNA-modified probes (double or single FAM-labeled) complementary to miR-96-5p and *FoxO1* mRNA were hybridized on consecutive 6 μm paraffin sections from G1, G2, and G3 GEP-NETs patients. An additional section was stained with H&E to reveal the overall morphology **(A–C)**. miR-96-5p was predominantly localized in the cytoplasm in all grades and its expression increased from G1 to G3 **(D–F)**. Inversely, *FoxO1* expression was localized in the nucleus in G1 and G2 sections and in the cytoplasm in G3 **(G–I)**. Original magnification, ×20. HE, hematoxylin and eosin stain.

The role of miR-96-5p has been extensively studied in various type of tumors, whereas no studies have yet addressed miR-96-5p expression in GEP-NETs tumor grades. miR-96-5p was found to be abnormally expressed in colorectal cancer, prostate cancer, and several other malignant tumors (41–44) where miR-96-5p can regulate *FoxO1* levels and consequently inhibit proliferation (45). To confirm the relationship between miR-96-5p and *FoxO1* expression in GEP-NETs, we studied their expression and localization in an independent cohort of different grade GEP-NETs patients by *in situ* hybridization. In particular, miR-96-5p was predominantly localized in the cytoplasm in all grades and showed increasing expression from G1 to G3 (Figure 4). By contrast, *FoxO1* expression was localized in the nucleus in G1 and G2 sections and in the cytoplasm in G3. Contrariwise to miR-96-5p, the expression of *FoxO1* was decreased from G1 to G3 (Figure 4).

### FoxO1 Protein Expression in GEP-NETs

**Figure 5:**
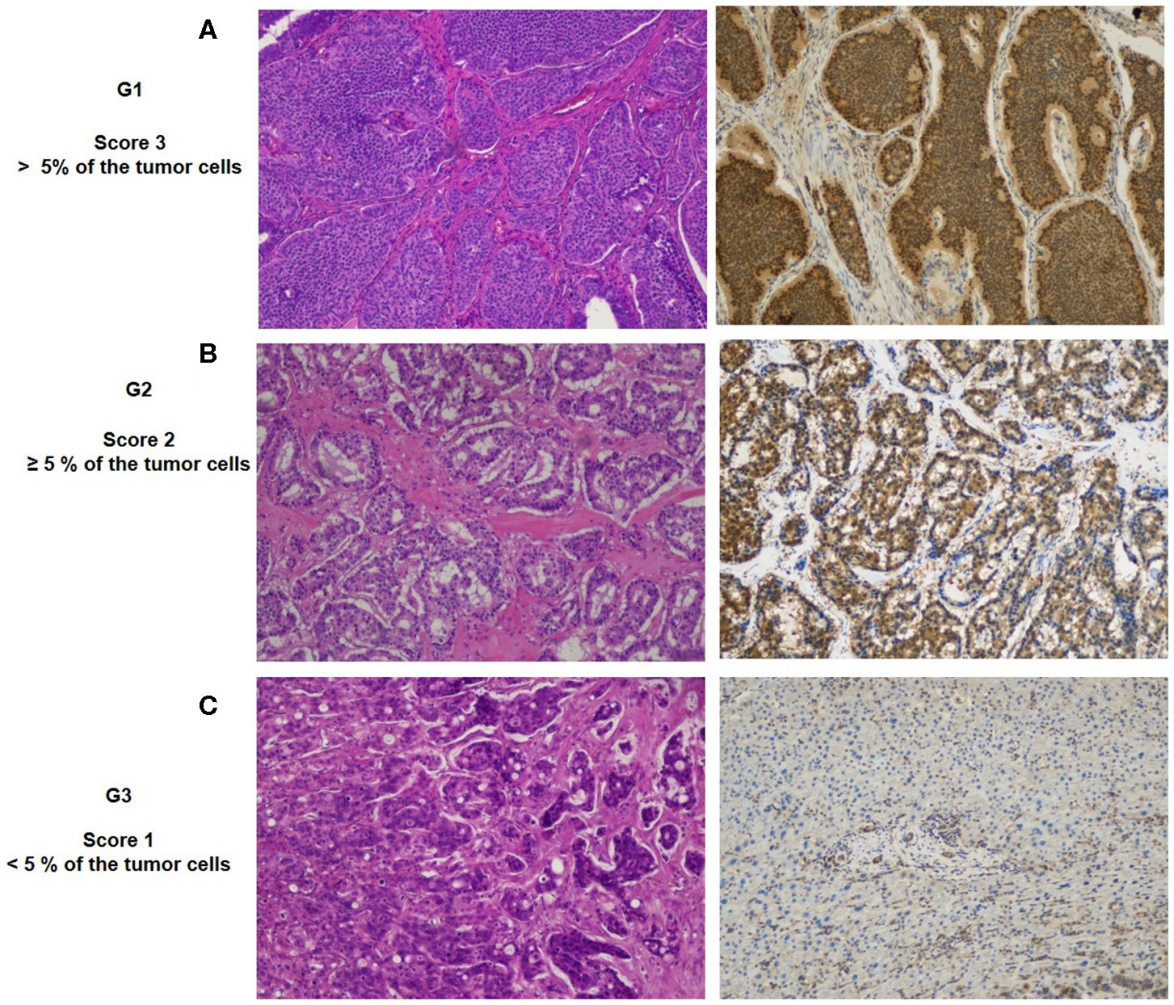
Representative patterns of FoxO1a staining intensity and grade: **(A)** G1, IHC strong expression; **(B)** G2, medium expression; **(C)** G3, weak expression. Original magnification, ×20.

To evaluate the biological significance of miRNA associated with G3 NETs, we analyzed the FoxO1 tissue expression in 90 GEP-NETs tissue by IHC (Figure 5). Among them, 78 cases resulted positive (86.7%) and 12 cases were negative (13.3%). The staining highlighted FoxO1 in the cytoplasm or in the nucleus. The relationship between FoxO1 expression and patients' gender, age, as well as tumor size and lymph node metastasis status, was not significant (data not shown). Notably, we highlighted a significant correlation between the protein expression of FoxO1 and grading of GEP-NETs (*p* = 0.001). In particular, the FoxO1 staining intensity score on neoplastic cells was different among the three grades (Figures 5A–C). Based on the FoxO1 signal intensity, we created a score (Table 2) from 0 to 3+ (absent to strong). In particular, G1 patients showed no signal in one tumor (1.61%), a weak signal in 9 (14.5%), moderate signal in 13 tumors (21%) and strong signal in 39 tumors (63%). FoxO1 expression was absent in 11 (68.7%) G3 patients while in G2 FoxO1 expression was present in all cases, although with a different intensity: weak staining in 1 (8.3%) cases, medium in 4 (33.3%) and strong in 7 (58.4%) cases (Table 2). Importantly, only one sample (6.3%) of G3 cases showed moderate positivity and cytoplasmic staining. Instead, 58.4% of G2 cases showed strong positivity and nuclear staining. Therefore, there is a direct correlation between FoxO1 signal intensity and GEP-NETs grading from G1 to G3 (*p* = 0.001) (Table 2).

**Table 2:** FoxO1a expression on different grade of GEP-NET.

	**Grade**			
	**G1**	**G2**	**G3**	*p*-value^*^
	**(*n* = 62)**	**(*n* = 12)**	**(*n* = 16)**	
**Score FoxO1(%)**				<0.001
Absent	1 (1.61)	0 (0.00)	11 (68.7)	
Weak	9 (14.5)	1 (8.3)	4 (25.0)	
Moderate	13 (21.0)	4 (33.3)	1 (6.3)	
Strong	39 (63.0)	7 (58.4)	0 (0.00)	

* *Refers to the statistical significance of the comparison*.

## Discussion

GEP-NETs are a heterogeneous group of tumors with a common phenotype but different origin, morphology, function, type and aggressiveness of the specific site prognosis and response to treatment. In these neoplasms, the histological evaluation is a crucial element in clinical management. Currently, Ki-67 staining and mitotic counts, is considered the most reliable predictor for tumor grading (46). This scoring method is poorly reproducible and time-consuming (47), thus there is an urgent need for novel approaches to support histological evaluation and prognosis.

miRNAs, a class of small non-coding RNAs, are recognized as key regulators of gene expression involved in diverse biological processes. Dysregulation of miRNA has been shown to play an important role in many cancers (48). Due to the specificity and stability of miRNAs in FFPE tissues, the assessment of miRNAs expression represents a promising novel approach to subclassify some tumors (49). Currently available data regarding miRNAs expression in GEP-NETs are limited since many studies has been conducted in pNETs (26–29). Some studies evaluated the clinical utility of miRNA as biomarkers in GEP-NETs and assessed their expression in different tissue and blood but to date no one highlights their expression in different grades of GEP-NETs. In particular, Panarelli N. et al. classified accurately pathological types of GEP-NET based on miRNA expression and constructed a double hierarchical classifier that firstly separates midgut from non-midgut NETs based on miR-615 and miR-92b expression. In the second, the classifier differentiated ileal from appendiceal NETs based on miR-125b, miR-192, and miR-149 expression, and rectal from pancreatic NETs based on miR-429 and miR-487b expression (50). Another interesting study, after a systematic literature overview of dysregulated miRNAs in GEP-NET, identified miR-21 as a potential marker for small bowel and pancreas NETs, although this marker required prospective (23).

In the present study, we defined the miRNAs signature for poorly differentiated NETs compared to well-differentiated NETs identifying 56 deregulated miRNAs. Moreover, for the first time, we found 8 miRNAs that were expressed in all GEP-NETs grades (miR-10b-5p, miR-130b-3p, miR-192-5p, miR-194-5p, miR-210-3p, miR-214-3p, miR-7-5p, and miR-96-5p), but their expression level was different between GEP-NETs grades. Among these miRNAs, we focused our attention to miR-96-5p that raised its expression levels from grade 1 to grade 3. Several studies have demonstrated that miR-96 was remarkably increased in several different types of cancers, such as pancreatic cancer, lung cancer, osteosarcoma, and gastric carcinoma, suggesting the expression of miR-96 was associated with the progression of tumor (51–53). In GEP-NET, we demonstrated that FoxO1 expression was markedly decreased in G3 patients, but was substantially higher in G1 patients.

FoxO1a is a Forkhead box O (FOXO) transcription factor and a downstream target of the IGF-1R/PI3K/Akt pathway implicated in several physiological and pathological processes including cancer. In malignancies, FoxO1 was shown to be an important tumor suppressor gene and was downregulated in many types of tumors (54). However, the molecular mechanism resulting in FoxO1 aberrant expression is poorly understood and the role of FoxO1 in tumorigenesis is not entirely clear. It has been hypothesized that the downregulation of this gene is an important step in tumor formation (55, 56). FoxO1 activity and function is regulated through shuttling between the nucleus and the cytoplasm (57). The nuclear active form of FoxO1 mediates the transcription of a broad array of target genes implicate in apoptosis, redox homeostasis, cell cycle inhibition, angiogenesis, and metabolism (58).

Several points of evidence suggest that the FoxO family of transcription factors are regulated by miRNAs. In prostate cancer, FoxO1 was regulated by miR-96, promoting cancer progression (59). In breast cancer cells, FoxO1 expression was directly regulated by three miRNAs (miR-27a, miR-96, and miR-182). The inhibition of these mRNAs led to the restoration of FoxO1 expression that in turn contributes to the transformation or maintenance of an oncogenic state in breast cancer cells (55). A recent study described that miR-96 was up-regulated in HCC tissues and HepG2 cells, miR-96 inhibiting FoxO1 and thus activating the AKT/GSK-3β/β-catenin signaling pathway, exerted its carcinogenic effect (60).

In our study, we demonstrated that in poorly differentiated GEP-NETs, miR-96-5p expression is upregulated and FoxO1 downregulated, suggesting a potential direct involvement of miR-96-5p in regulating FoxO1 expression. On the contrary, in well-differentiated G1 GEP-NETs patients miR-96-5p expression was lower and FoxO1 positive cells were more frequent. Furthermore, we localized miR-96-5p and FoxO1 expression, finding that miR-96-5p was predominantly localized in the cytoplasm in all tumor grades and its expression increasing from G1 to G3. Interestingly, FoxO1 expression was localized in the nucleus in G1 and G2 section and in the cytoplasm in G3. Likewise, analysis of FoxO1 at protein levels showed that in 68.7% of G3 patients FoxO1 signal was absent and the remaining part of G3 cases showed weak positivity and cytoplasmic localization. Instead, the 63% of G1 patients showed strong positivity and nuclear staining.

Our data are in line with data reported by several previous studies suggesting that low expression of the FoxO family is correlated with poor clinical outcomes in several cancers (57, 61). Similarly, other studies reported a correlation among high FoxO expression, nuclear localization and good prognosis (62). Here, we have shown a significant negative correlation between FoxO1 expression and grading, whereby well-differentiated NETs (G1-G2) cases have a persistent positive FoxO1 nuclear/cytoplasmatic expression that decreases with the tumor progression, confirming the more favorable outcomes in patients G1/G2 GEP-NETs.

In addition, the effect of FoxO1 activity on chemosensitization has been demonstrated in several tumors (63). Our data on FoxO1 absence in poorly differentiated GEP-NET patients could, at least partially explain the chemoresistance observed in this group of patients (64).

In conclusion, our study explored the miRNA expression profile of GEP-NETs, correlating their expression with grading. We defined the specific miRNA signature for each GEP-NET grade identifying miRNAs that were commonly expressed in all GEP-NET but at different levels. In particular, we demonstrated that miR-96-5p expression level was higher in grade 3 GEP-NETs. In line with this observation, we proved that the miR-96-5p target FoxO1 was poorly expressed in G3 GEP-NETs. Furthermore, FoxO1 nuclear expression was detected in G1 and G2 GEP-NETs, in line with a more favorable prognosis of these patients. Altogether, our results indicate a potential advantage of miRNAs quantification to aid clinicians in the classification of common GEP-NETs subtypes. Therefore, the combination of conventional strategies implemented with miRNAs expression and FoxO1 histological assessment may represent a new gold standard to for GEP-NETs evaluation. Furthermore, miRNAs represent an innovative target for personalized treatment of several disease, but further studies on multicenter larger cohorts are needed to confirm and validate their potential as NETs markers. Our data demonstrate an important and unreported role of miRNAs as biomarker in GEP-NETs grading and suggest further investigation to address their therapeutic potentials.

## Data Availability Statement

The datasets generated for this study can be found in the Gene Expression Omnibus (GEO, https://www.ncbi.nlm.nih.gov/geo/query/acc.cgi?acc=GSE135034).

## Ethics Statement

The studies involving human participants were reviewed and approved by Ethics Committee of the Istituto Tumori Giovanni Paolo II (BA, Italy). The patients/participants provided their written informed consent to participate in this study.

## Author Contributions

EC and GS conceived and designed the project. EC, VG, SC, MC, and GS carried out the experiments. GS performed bioinformatic and statistical analysis. ES and RA performed the clinical and histological evaluations. EC and GS wrote the paper. All authors contributed to the article and approved the submitted version.

## Conflict of Interest

The authors declare that the research was conducted in the absence of any commercial or financial relationships that could be construed as a potential conflict of interest.

## Supplementary Material

Supplementary Table 1 miRNAs differentially expressed in the comparison between patients with grading G3 (poorly differentiated NETs) vs G1 and G2 (well differentiated NETs).

Supplementary Table 2 Top rank pathways regulated by miRNAs.

Supplementary Table 3 Significant Gene Ontology biological processes regulated by miRNAs.

## Abbreviations

GEP-NETs: Gastroenteropancreatic neuroendocrine tumors
NETs: Neuroendocrine tumors
miRNA: microRNA
PD-L1: programmed cell death ligand 1
H&E: Hematoxylin and eosin
FFPE: formalin fixed paraffin embedded
IHC: Immunohistochemistry
DMSO: Dimethyl sulfoxide
FDR: false discovery rate
DAPI: 4′,6-diamidino-2-phenylindole.
